# A paired dataset of multi-modal MRI at 3 Tesla and 7 Tesla with manual hippocampal subfield segmentations

**DOI:** 10.1038/s41597-025-04586-9

**Published:** 2025-02-13

**Authors:** Lei Chu, Baoqiang Ma, Xiaoxi Dong, Yirong He, Tongtong Che, Debin Zeng, Zihao Zhang, Shuyu Li

**Affiliations:** 1 Beijing Advanced Innovation Center for Biomedical Engineering, School of Biological Science & Medical Engineering, Beihang University, Beijing, 100083 China; 2 Department of Radiation Oncology, University Medical Center Groningen, University of Groningen, Groningen, the Netherlands; 3 State Key Laboratory of Cognitive Neuroscience and Learning, Beijing Normal University, Beijing, 100875 China; 4 State Key Laboratory of Brain and Cognitive Science, Institute of Biophysics, Chinese Academy of Sciences, Beijing, 100101 China; 5 Anhui Province Key Laboratory of Biomedical Imaging and Intelligent Processing, Institute of Artificial Intelligence, Hefei Comprehensive National Science Center, Hefei, 230088 China; 6 University of the Chinese Academy of Sciences, Beijing, 100049 China

**Keywords:** Brain, Medical research

## Abstract

The hippocampus plays a critical role in memory and is prone to neural degenerative diseases. Its complex structure and distinct subfields pose challenges for automatic segmentation in 3 T MRI because of its limited resolution and contrast. While 7 T MRI offers superior anatomical details and better gray-white matter contrast, aiding in clearer differentiation of hippocampal structures, its use is restricted by high costs. To bridge this gap, algorithms synthesizing 7T-like images from 3 T scans are being developed, requiring paired datasets for training. However, the scarcity of such high-quality paired datasets, particularly those with manual hippocampal subfield segmentations as ground truth, hinders progress. Herein, we introduce a dataset comprising paired 3 T and 7 T MRI scans from 20 healthy volunteers, with manual hippocampal subfield annotations on 7 T T2-weighted images. This dataset is designed to support the development and evaluation of both 3T-to-7T MR image synthesis models and automated hippocampal segmentation algorithms on 3 T images. We assessed the image quality using MRIQC. The dataset is freely accessible on the Figshare+.

## Background & Summary

The hippocampus plays an important role in clinical neuroscience due to its critical functions in long-term episodic memory consolidation, spatial navigation, and learning^1–3^. Previous studies have demonstrated that the hippocampus is not a uniform structure; rather, it is comprised of distinct subfields, each exhibiting unique functional profiles, connectivity patterns, and differential vulnerabilities to specific disease processes^4,5^. Precise delineation of hippocampus subfields is crucial for the diagnosis and management of various neurological and psychiatric disorders. Moreover, the picture is made more complex by the fact that the hippocampus is not a homogeneous brain structure. It is composed of two convoluted structures: the dentate gyrus (DG) and the Cornu Ammonis (CA), with the latter divided into four subdivisions (CA1–4). The subiculum (SUB) is also typically recognized as a component of the hippocampal complex. In conjunction with the entorhinal cortex (ERC), these elements constitute the hippocampal formation^6^. Despite the availability of automated methods for hippocampal subfield segmentation, such as FreeSurfer v6.0^7^, ASHS^8^, MAGeT-Brain^9^, HIPS^10^, CAST^11^, HippUnfold^12^, HSF^13^, the performance of these supervised methods largely depend on the accuracy of manual segmentation, which serves as the gold standard. Such manual segmentations require substantial expertise and a considerable time investment—approximately eight hours per subject when executed by an experienced delineator^14^. Therefore, researchers face a notable scarcity of databases providing comprehensive manuals for hippocampal subfield segmentation to guide the development of advanced and automated segmentation methods.

Further compounding the challenge is the inherent limitation of standard 3 T MRI in resolving the fine anatomical details of hippocampal subfields due to limited signal contrast and resolution. In contrast, 7 T MRI, with its superior spatial resolution and signal-to-noise ratio (SNR), provides significantly enhanced anatomical detail^15,16^, improving the accuracy of various neuroimaging post-processing tasks, including tissue segmentation and cortical surface reconstruction. The ability of 7 T MRI to reveal subtle pathological changes invisible on lower-field-strength scanners further enhances its value in both research and clinical diagnostics^17–19^. Additionally, imaging techniques that utilize susceptibility-induced contrast, including functional MRI (Blood Oxygenation Level Dependent, BOLD), which benefit greatly from the ultra-high field strength^20^, enabling the construction of mechanistic models of cognitive function^21^. Even in the hippocampus, where primary structures within the hippocampal formation are visible at clinical 3 T MR scanner, encounters resolution limitations that impede the delineation of finer subfield anatomy and distinct signal profiles achievable at higher field strengths^22^. This is especially critical considering the hippocampus’s small volume (typically measuring 1–3 ml in elderly subjects, including those with Alzheimer’s disease) which is represented by only 1000–3000 voxels at the standard clinical resolution of 1 × 1 × 1 mm^3^. Moreover, with 80–90% of these voxels located on the surface, partial volume effects can substantially impact volume estimation accuracy^23^. 7 T MRI substantially mitigates these partial volume effects, revealing more anatomical details^24^ and improving the interpretation of imaging data acquired at standard clinical field strengths^25^. The superior SNR and ultra-high resolution of 7 T MRI enable the consistent visualization of internal features while maintaining a thin slice thickness (as fine as 1 mm), essential for revealing the intricate anatomy of the hippocampal head. Notably, high-resolution T2-weighted images at 7 T have demonstrated their efficacy in demarcating medial temporal lobe (MTL) subregions via the vivid delineation of the stratum radiatum lacunosum moleculare (SRLM), which manifests as a distinct dark band and acts as a critical marker for demarcating subregion boundaries^26^.

Despite their significant benefits in improving tissue contrast and anatomical detail, the integration of 7 T MRI scanners in research and clinical settings is primarily restricted by high cost and complex maintenance demands^27^. To circumvent these challenges and enhance the utility of the more prevalent 3 T MRI datasets, various deep learning-based 3T-to-7T image synthesis methods have been proposed, demonstrating promise in generating realistic high-resolution 7T-like images from 3 T scans^27–29^. However, for super-resolution task, obtaining a paired dataset that includes both low and high-resolution MRIs for algorithm training constitutes a practical hurdle considering most existing approaches are implemented on private datasets^30^. While several Generative Adversarial Networks (GANs) based synthesis models can be trained in the absence of paired images, paired data remains crucial for quantitative model validation^31^.

Here, we disseminate a dataset of whole-brain paired T1-weighted, T2-weighted and resting-state fMRI (rfMRI) scans acquired at 3 T and 7 T scanners, featuring manual hippocampal subfield annotations on the 7 T T2-weighted images. We provide a comprehensive description of the design, acquisition, and preparation of the dataset. Image quality is assessed using quality metrics implemented in MRIQC^32^. This dataset has already been utilized to develop Syn_SegNet^33^, an end-to-end, multitask joint deep neural network that leverages 7 T MRI synthesis to improve hippocampal subfield segmentation in 3 T MRI. We anticipate that this resource will significantly advance the field by facilitating research and development in 3T-to-7T image synthesis, T1-to-T2 image translation, and functional MRI analysis, improving the accuracy and accessibility of hippocampal subfield segmentation.

## Methods

### Participant recruitment

Twenty healthy volunteers (10 males and 10 females) aged 18–25 years were recruited from university. Inclusion criteria were: 1) no history of brain injury, neurological, or psychiatric disorders; 2) a Mini-Mental State Examination (MMSE)^34^ score ≥ 24; 3) absence of contraindications to MRI scanners, such as claustrophobia or the presence of metallic implants. All participants provided written informed consent, including permission for open sharing of anonymized data. Ethical approval was granted by the Science and Ethics Committee of the School of Biological Science and Medical Engineering in Beihang University (Application No. 20140304), in accordance with the Declaration of Helsinki.

Additional demographic information collected included handedness (all participants were right hand dominant, confirmed using the Edinburgh Handedness Inventory^35^) and years of education (mean = 17.25 years, SD = 0.69).

Ten questionnaires were administered in the following order: Basic Information Questionnaire, Edinburgh Handedness Inventory (EHI), MMSE, Self-Rating Depression Scale (SDS)^36^, Self-Rating Anxiety Scale (SAS)^37^, Pittsburgh sleep quality index (PSQI)^38^, Wechsler Adults Intelligence Scale (WAIS-RC)^39^, Raven’s Progression Matrices (RPM), The Schutte Self Report Emotional Intelligence Test (SSEIT)^40^, and The Trail Making Test (TMT)^41^. Detailed demographic and questionnaire data are presented in Table 1.

**Table 1:** Demographic information and questionnaire results of the 20 participants.

id	sex	age	years of education	weight (kg)	height (m)	BMI	SDS	MMSE	SAS	PSQI	EI	WAIS-operation	WAIS-language	WAIS-RC	RPM	TMT-A (s)	TMT-B (s)
sub-01	female	24	17	47	1.62	17.91	28	30	33	4	142	122	116	120	52	26	106
sub-02	female	22	17	49	1.54	20.66	25	30	34	2	103	131	128	132	59	25	48
sub-03	male	24	18	56	1.77	17.87	35	30	35	14	95	117	133	129	57	37	86
sub-04	female	23	17	53	1.61	20.45	31	30	38	2	115	109	123	118	59	53	76
sub-05	female	21	16	52	1.65	19.10	27	30	28	2	116	131	132	135	60	31	42
sub-06	female	25	18	49	1.70	16.96	28	30	36	1	146	127	127	129	58	27	51
sub-07	male	26	17	67	1.81	20.45	40	30	30	12	87	122	128	128	60	26	55
sub-08	male	22	17	80	1.75	26.12	30	30	34	5	133	120	136	132	58	22	35
sub-09	male	23	16	66	1.74	21.80	30	30	34	5	120	119	132	129	57	31	53
sub-10	male	24	17	95	1.80	29.30	31	30	35	6	134	130	127	130	60	21	62
sub-11	male	22	19	63	1.74	20.81	34	30	38	10	115	130	138	137	60	23	44
sub-12	male	23	17	78	1.83	23.30	26	28	34	8	122	114	114	114	52	38	52
sub-13	female	21	17	49	1.60	19.14	30	30	40	5	115	110	115	114	56	29	52
sub-14	female	23	17	50	1.68	17.72	34	30	33	5	112	117	125	123	57	25	60
sub-15	female	24	18	80	1.74	26.40	31	30	35	6	126	131	127	131	58	24	46
sub-16	female	25	18	48	1.60	22.80	31	30	32	5	149	128	126	129	55	39	63
sub-17	female	23	17	56	1.65	20.57	33	30	36	11	109	131	127	131	58	67	38
sub-18	male	22	17	65	1.77	20.75	31	30	37	1	137	116	135	128	55	43	83
sub-19	male	25	17	66	1.70	22.84	26	30	32	5	123	129	136	136	59	40	83
sub-20	male	25	18	74	1.75	24.20	29	30	22	1	128	112	119	116	52	71	133

Edinburgh Handedness Inventory (EHI): Determined handedness, crucial for interpreting lateralized brain functions. Mini-Mental State Examination (MMSE): Screened for cognitive impairment, ensuring a cognitively healthy sample. Self-Rating Depression Scale (SDS): Assessed depressive symptoms, which can influence brain structure and function. Self-Rating Anxiety Scale (SAS): Measured anxiety levels, another factor that can affect neuroimaging results. Pittsburgh Sleep Quality Index (PSQI): Evaluated sleep quality, known to impact cognitive performance and brain health. Wechsler Adults Intelligence Scale (WAIS-RC): Provided a comprehensive measure of cognitive abilities. Raven’s Progressive Matrices (RPM): Assessed non-verbal reasoning skills. Schutte Self-Report Emotional Intelligence Test (SSEIT): Measured emotional intelligence, relevant to social cognition studies. Trail Making Test (TMT): Evaluated executive function and cognitive flexibility

.

### Image acquisition

MRI data were acquired from each participant using both a 3 T Prisma and a 7 T MAGNETOM MR scanner (Siemens Healthineers, Erlangen, Germany) on the same day at the Beijing MRI Center for Brain Research (BMCBR). A 32-channel receive and single-channel transmit head coil (Nova Medical, Wilmington, MA, USA) was used for 7 T scans, and a 64-channel phased-array head coil (Siemens Healthineers, Erlangen, Germany) was used for 3 T scans.

### 3 T sequences

T2-weighted turbo spin echo (TSE) sequence was obtained with a resolution of 0.9 × 0.9 × 1.9 mm^3^. The sequence parameters were: repetition time (TR) = 11050 ms, echo time (TE) = 94 ms, flip angle (FA) = 60°, bandwidth = 100 Hz/Px, and acquisition matrix size = 256 × 256 × 90. One volume acquired perpendicular to the hippocampus long axis.

An isotropic 1mm^3^ T1-weighted sagittal 3D magnetization-prepared rapid gradient echo (MPRAGE) sequence was acquired along the long axis of the hippocampus, with the following settings: TR = 2300 ms, inversion time (TI) = 1000 ms, TE = 2.26 ms, FA = 8°, and acquisition matrix size = 256 × 224 × 192.

Whole-brain resting-state fMRI was obtained using a 2D echo-planar imaging (EPI) sequence with the following scanning parameters: FA = 50°, TR = 600 ms, TE = 30 ms, resolution = 3.0 × 3.0 × 3.0 mm^3^, and acquisition matrix size = 80 × 80 × 48. One run with a total of 800 volumes was acquired.

The diffusion-weighted imaging (DWI) scan was acquired using a 2D EPI sequence with a slice thickness of 1.3 mm and the same spacing between slices. The parameters were: TR = 5500 ms, TE = 82 ms, and flip angle = 90°. The acquisition included a multiband acceleration factor of 3 and a parallel reduction factor in-plane of 2. The base resolution and acquisition matrix were both 162, with an echo train length of 61. The EPI factor used was 128.

### 7 T sequences

T2-weighted Sampling Perfection with Application-optimized Contrasts by using flip angle Evolution (SPACE) sequence: 3D, 0.4 × 0.4 × 1.0 mm³, TR = 3000 ms, TE = 387 ms, FA = 120°, bandwidth = 279 Hz/Px, matrix = 480 × 512 × 224. Two volumes acquired (with and without non-distortion correction, with and without a suffix of ‘ND’ in filename).

T1-weighted MPRAGE: Sagittal, isotropic 0.7 mm³, TR = 2600 ms, TI = 1050 ms, TE = 2.72 ms, FA = 5°, matrix = 320 × 320 × 256. Two volumes acquired (with and without non-distortion correction).

Resting-state fMRI (EPI): 3D, FA = 45°, TR = 1000 ms, TE = 21 ms, resolution = 1.5 × 1.5 × 1.5 mm³, matrix = 128 × 128 × 88. One run of 800 volumes.

### Segmentation of hippocampal subfields

**Fig. 1:**
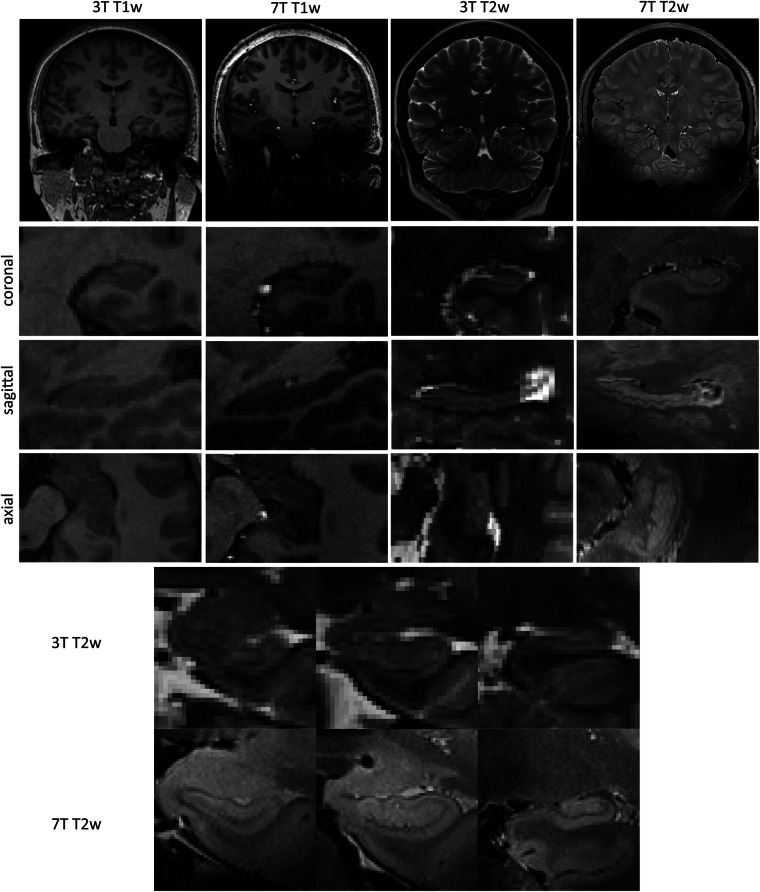
Paired T1-weighted and T2-weighted MRI scans at 3 T and 7 T acquired for a subject (sub-10), the left hippocampus of the subject was shown for multiple views and multiple modalities. The final two rows depict zoomed-in coronal views of the hippocampus from head to body at three different slices, comparing 3 T and 7 T T2-weighted images. The 3 T images show lower resolution and significant partial volume effects, while the 7 T images, with enhanced resolution, more clearly demonstrate the hippocampal internal architecture, outer boundaries, and inner boundaries between subfields, with the hypointense line appearing more consistent and visible compared to 3 T.

**Fig. 2:**
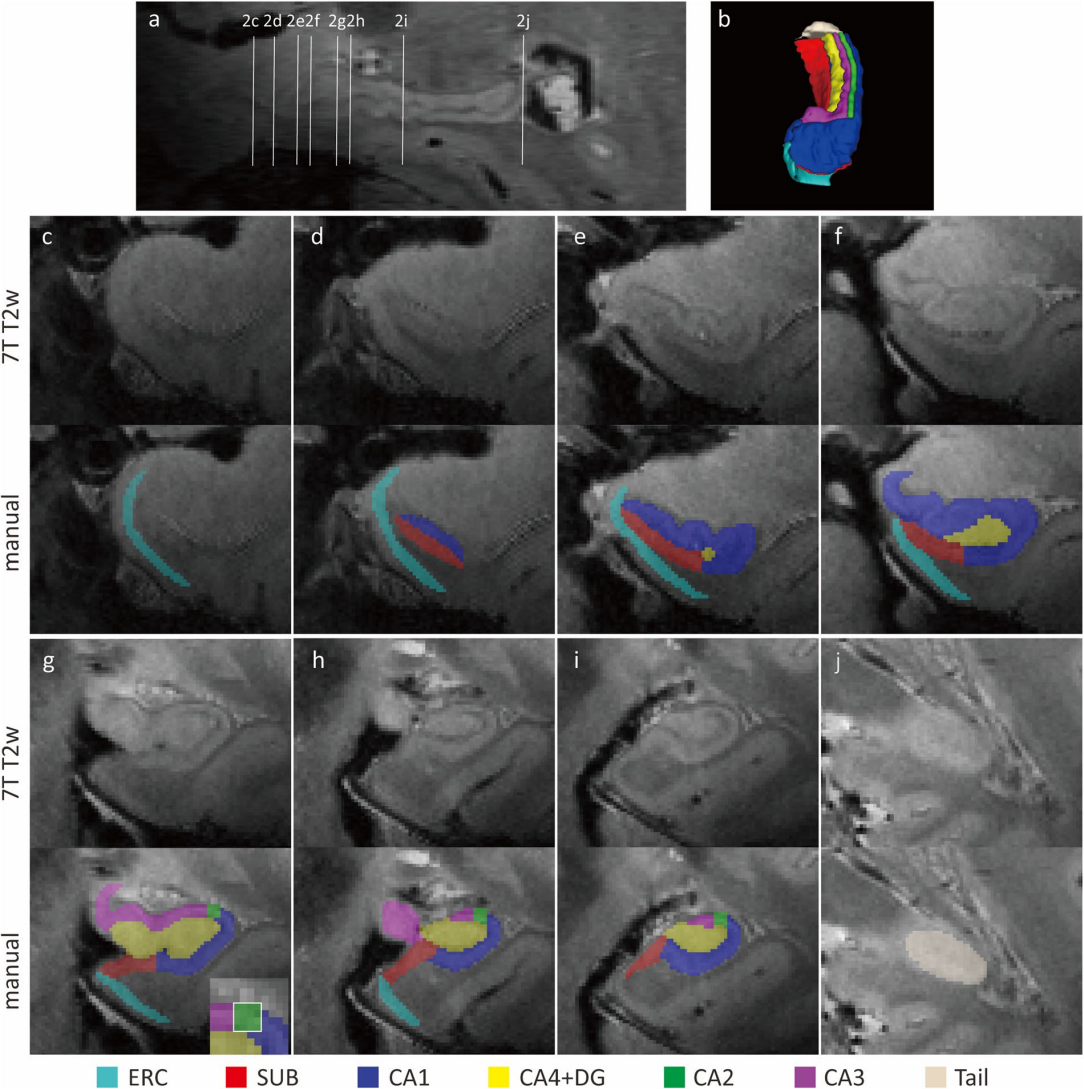
Manual segmentation of the hippocampal formation in subject 12. (**a**) A sagittal view illustrating references to the sequential coronal sections. Coronal images depicting the hippocampal formation and accompanying manual segmentation are presented from anterior to posterior, labeled as 2c-2j. (**b**) A 3D reconstruction viewed from an anterolateral angle highlights the segmented hippocampal subregions. (**c**–**j**) For each coronal section, the top row displays the 7 T T2-weighted images, while the bottom row depicts manual delineation of hippocampal subfields. A detailed zoom-in view (seen in image 1 g) demonstrates the delineation of the border between CA2 and CA3 by drawing a virtual square. Abbreviations: ERC, entorhinal cortex; SUB, subiculum; CA, Cornu Ammonis; DG, dentate gyrus.

We followed the protocol established by Wisse *et al*.^14^, which integrates elements from pre-existing methods with new rules to ensure coverage of all subfields throughout the entire length of the hippocampal formation. The hippocampal formation subfields, including the ERC, SUB, CA1, DG-CA4, CA2, CA3, and the hippocampal tail, were manually delineated. Segmentation was performed bilaterally on every coronal section of the subjects’ ultra-high-resolution 7 T T2-weighted MRI scans using ITK-SNAP software^42^ (version 3.8.0, www.itksnap.org), where 7 T T2-weighted images have been upsampled to a 0.7 mm slice thickness utilizing B-spline interpolation, after Gaussian smoothing denoising and N4 bias field correction with Advanced Normalization Tools (ANTs). Figure 1 displays MRI images from four different modalities of a single subject, whereas Fig. 2 illustrates the manual segmentation results of the left hippocampus alongside its corresponding 3D reconstruction.

It should be noted that the implementation of the original protocol required adaptations for use with ITK-SNAP software. Specifically, the delineation of hippocampal subfields was adjusted to accommodate ITK-SNAP’s voxel-by-voxel approach, without modifying the underlying protocol principles. This is in contrast to the freehand spline drawing technique described in the reference protocol by Wisse *et al*.^14^. The subfields were manually traced on coronal images from anterior-to-posterior, beginning with Fig. 2c and continuing to 2j on coronal images. The head of the hippocampus is depicted in Fig. 2d–h, the body in Fig. 2i, and the tail in Fig. 2j. These modifications are detailed below, following the sequence of annotation.

(i) The border between the SUB and CA1 in the most anterior slices of the hippocampus was determined by measuring the longest diameter from the medial to the lateral point (Fig. 2d,e). With the emergence of the DG, this border changed, and the SUB became laterally bordered by CA1 at the most medial point of the DG. Given the small size of the DG in early slices, a practical approach involved extrapolating backwards from subsequent levels where the DG was larger and examining the region from multiple perspectives, especially in the sagittal view.
(ii) The delineation of CA2 and CA3 subfields commenced at a position 1.4 mm anterior to the separation point of the uncus from the hippocampus on coronal images, where only the fimbria exhibited attachment to both structures as per the previous protocol. To ensure accuracy and consistency, delineation started on a slice located two slices ahead, assuming a slice thickness of 0.7 mm (Fig. 2g,h).
(iii) As outlined in the protocol^14^, the CA2-CA3 border was established by defining the medial side of a virtual square. This square was positioned with its lateral side touching the border of CA1 and its superior side aligned along the superior border of CA2. To ensure accuracy and consistency during the segmentation process using ITK-SNAP, a paintbrush size of 4 was employed. The delineation of the virtual square between CA2 and CA3 was accomplished using 4 × 4 voxel squares in most instances, as this size matched the thickness of CA2 at its boundary with CA3 (Fig. 2g).
(iv) The delineation of hippocampal subfields encompassed the entire length of the long axis of the hippocampal formation. However, due to the limited visibility of the total length of the fornix beyond a certain section, reliable delineation of fused subfields was not feasible. To address this limitation and maintain consistency, the last 4–6 slices were labeled as “Tail” (Fig. 2j) in accordance with a previously published protocol^14^.

## Data Records

The dataset is available at Figshare+^43^. The raw MRI data are stored in*.ima* format within the ‘*rawdata_DICOM_3T/7T’* directories. The rawdata DICOM 7 T data are further divided into four parts, each containing raw DICOM data for 5 subjects. Additionally, the acquired MRI scans have been converted from DICOM to the Neuroimaging Informatics Technology Initiative (NIfTI) format and organized in accordance with the Brain Imaging Data Structure (BIDS) format by employing the BIDScoin Python application (version 4.3.0)^44^. These files are stored in the ‘*rawdata_BIDS_3T/7T’* directories.

Hippocampal subfield segmentation related files can be found in the *‘hippo_subfield\7T_T2-weighted_0.7_for_subfield_delineation’* directory. The *‘hippo_subfield\hippo_label’* directory contains manual segmentation labels for seven hippocampal subregions for each subject. Detailed information on segmentation labels, including label values and colors for each hippocampal subfield is documented in *‘hippo_subfield\subfield label.txt’*.

The *‘MRIQC_3T/7T’* directories store the results of quality control assessments. For each participant, the ‘MRIQC_3T/7T\sub*’ directories contain *\anat* and *\func* subdirectories, which hold image quality metric reports for T1-weighted, T2-weighted, rfMRI scans. These quality metrics, available in both*.html* and*.json* formats, are instrumental in evaluating data quality and provide estimates of motion, SNR, and intensity non-uniformities, supplemented with visual reports in*.svg format* in the *‘\figure’* subdirectory.

**Fig. 3:**
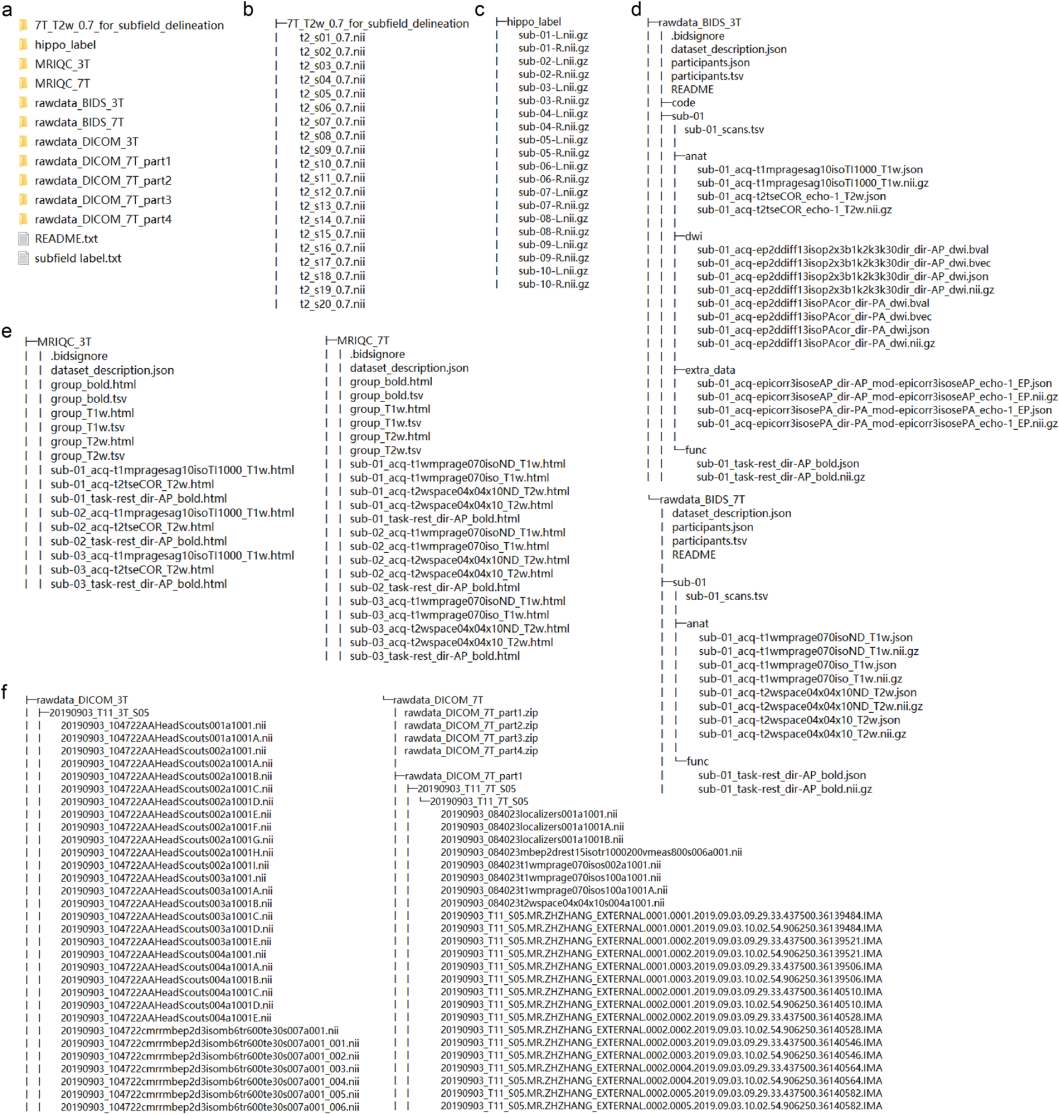
The directory structure of the dataset. (**a**) Overview of all the main folders included in the shared dataset. (**b**) The folder ‘T1_T2w_0.7_for_subfield_delineation’ contains 7 T T2-weighted images of each participant, which were used for hippocampal subfield delineation. (**c**) Contents of the ‘hippo_label’ folder, including subfield label files of each subject. (**d**) The directory structure for the 3T-7T paired dataset, conforming to BIDS standards. (**e**) Detailed image quality reports for T1-weighted, T2-weighted, and resting-state fMRI data, generated with MRIQC, are included. (**f**) The ‘*rawdata_DICOM_3T’* and ‘*rawdata_DICOM_7T’* folders contain anonymized DICOM data for 3 T and 7 T, respectively, without additional processing.

Notably, due to heavy head motion during the first scans, the 3T T2-weighted images for two participants (sub 06 and 12) were rescanned. Only the datasets from these subsequent sessions are retained in the *‘rawdata\BIDS’* directory for further quality evaluation. Figure 3 illustrates the directory structure of our dataset.

## Technical Validation

### Hippocampal subfield segmentation on 7 T T2-weighted high resolution structural images

High-resolution 7T T2-weighted structural images were acquired to enable precise manual segmentation of hippocampal subfields. High image quality ensured the visibility of crucial anatomical landmarks, facilitating accurate delineation in all participants. To assess inter- and intra-rater reliability, two experienced medical experts (L.C. and X.D.) independently segmented the hippocampal subfields of ten subjects (50% of the sample) on two separate occasions, one month apart. This procedure established proficiency with the segmentation protocol. Subsequently, these raters collaboratively produced the final manual segmentation for all twenty subjects, reaching consensus on any discrepancies through discussion.

Reliability of the hippocampal subfield segmentations was evaluated using inter- and intra-rater reliability measures: Intraclass Correlation Coefficients (ICC)^45^ and Dice Similarity Coefficients (DSC)^46^. These metrics were calculated for each subfield, separately for the left hemisphere, right hippocampus, and bilaterally.

Intra-rater reliability, reflecting the consistency of individual rater performance, was evaluated using both ICCs and DSCs. ICCs were calculated as:

$${\rm{ICC}}=\frac{M{S}_{\text{R}}-M{S}_{\text{E}}}{M{S}_{\text{R}}+(M{S}_{\text{C}}-M{S}_{\text{E}})/n}$$

Where MS_R_ represents the mean square between subjects. MS_E_ (mean square error) represents the unexplained variance attributable to random error. MS_C_ represents the mean square between the two independent tracing sessions. n represents the number of subjects. DSCs were calculated as:

$$\mathrm{DSC}=\frac{2\times |X\cap Y|}{|X|+|Y|}$$

where X and Y represent the voxel sets from the two tracing sessions for a given subject, |X ∩ Y| denotes the number of overlapping voxels, and |X| and |Y| represent the total number of voxels in each segmentation. For intra-rater reliability, both ICCs and DSCs ranged from 0 to 1, with higher values indicating greater reliability. We considered ICC values of 0.75–0.90 as good, and values ≥ 0.90 as excellent.

Inter-rater reliability, reflecting the agreement between the two independent raters, was similarly assessed using ICCs and DSCs. The ICC calculation remained consistent, with MS_C_ now representing the mean square between the two raters. The DSC calculation also remained unchanged. For inter-rater reliability, both ICCs and DSCs ranged from 0 to 1, with higher values indicating greater agreement.

Table 2 presents the inter- and intra-rater reliability analyses. The results align with those of the reference protocol^14^. For intra-rater reliability, our enhanced analysis yielded a DSC of 0.810 for the ERC segment, slightly below the 0.83 reported by Wisse *et al*.^14^. Similarly, the CA2 segment’s DSC was 0.80, marginally lower than the 0.83 reported by Wisse *et al*.^14^. Conversely, our DSC values for the CA1 and SUB segments(0.875, respectively) closely matched the 0.85 and 0.84 values reported by Wisse *et al*.^14^ Regarding intra-rater ICCs, our scores for the ERC, SUB, and CA1 segments are 0.78, 0.96, and 0.97, respectively, marginally below the 0.80, 0.97, and 0.98 reported by Wisse *et al*.^14^ The inter-rater reliability findings mirrored the intra-rater results but exhibited slightly lower values.

**Table 2:** ICC and DSI of manual segmentations, intra-rater reliability of a single manual rater (L.C.), and interrater reliability of 2 independent manual raters.

	Mean inter-rater reliability						Mean intra-rater reliability					
	DSC (mean ± sd)			ICC			DSC (mean ± sd)			ICC		
	Left	Right	Combined	Left	Right	Combined	Left	Right	Combined	Left	Right	Combined
ERC	0.792 ± 0.023	0.800 ± 0.022	0.795 ± 0.020	0.75	0.77	0.74	0.805 ± 0.021	0.800 ± 0.019	0.810 ± 0.020	0.79	0.81	0.78
SUB	0.858 ± 0.064	0.865 ± 0.060	0.860 ± 0.062	0.95	0.94	0.94	0.870 ± 0.059	0.865 ± 0.061	0.875 ± 0.058	0.97	0.96	0.96
CA1	0.859 ± 0.021	0.865 ± 0.018	0.861 ± 0.019	0.93	0.95	0.95	0.870 ± 0.017	0.865 ± 0.020	0.875 ± 0.018	0.95	0.97	0.97
CA4&DG	0.882 ± 0.023	0.890 ± 0.021	0.884 ± 0.023	0.93	0.95	0.94	0.895 ± 0.020	0.890 ± 0.022	0.900 ± 0.021	0.95	0.97	0.96
CA2	0.780 ± 0.046	0.785 ± 0.045	0.780 ± 0.048	0.81	0.73	0.78	0.790 ± 0.044	0.785 ± 0.043	0.795 ± 0.042	0.84	0.76	0.81
CA3	0.785 ± 0.050	0.795 ± 0.050	0.788 ± 0.046	0.78	0.80	0.79	0.800 ± 0.049	0.795 ± 0.045	0.805 ± 0.048	0.81	0.83	0.82
Tail	0.733 ± 0.115	0.750 ± 0.110	0.745 ± 0.114	0.76	0.77	0.77	0.755 ± 0.109	0.760 ± 0.112	0.765 ± 0.107	0.79	0.80	0.80

ICC = Intraclass Correlation Coefficients, DSC = Dice Similarity Coefficient.

### Image quality metrics (IQMs) for MRI data

**Fig. 4:**
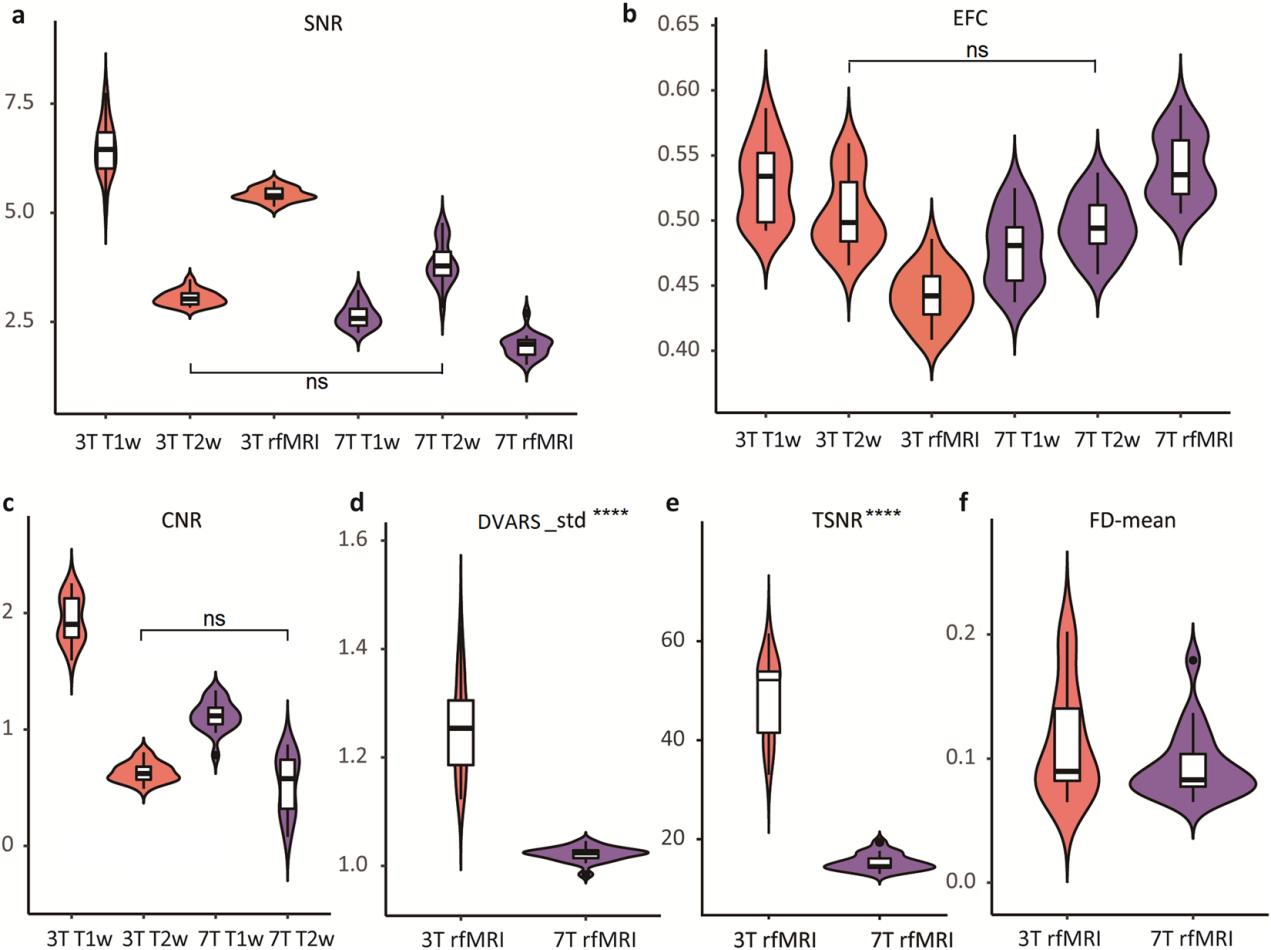
Violin plots illustrating the distribution of the six Image Quality Metrics (IQMs) across different acquisition protocols. Metrics include (**a**) Signal-to-Noise Ratio (SNR); (**b**) Entropy Focus Criterion (EFC); (**c**) Contrast-to-Noise Ratio (CNR); (**d**) Standardized DVARS (DVARS_std). (**e**) Total Signal-to-Noise Ratio (tSNR); and (**f**) Mean Framewise Displacement (FD-mean). Data collected at 3 T is shown in orange, while 7 T data is shown in purple. Asterisks (****) indicate that the p-value of the group difference is less than 0.001, while ‘ns’ denotes non-significant differences. For panels (**a**–**c**), statistically significant differences between 3 T and 7 T data are observed, except for T2-weighted images. The FD-mean metric shows no significant group difference between 3T and 7T resting-state functional MRI acquisitions.

All participants underwent an initial quality assessment of their neuroimaging data. We ensured that the datasets conformed to the BIDS standard using the BIDS-validator (version 1.14.3; available at https://github.com/bids-standard/bids-validator). Prior to quality assessment of the T1-weighted, T2-weighted, and rfMRI sequences, we used MRIQC^32^ to generate subject-specific visual quality assessment reports. These reports facilitated a comprehensive evaluation of processing quality. Detailed IQMs, calculated for all structural and functional imaging series using MRIQC are included in this data release. Six representative IQMs, presented in Fig. 4, are detailed below.

**Spatial anatomical and functional Metrics:**

(i) Signal-to-Noise Ratio (SNR)^47^: SNR was calculated within each tissue mask including gray matter (GM), white matter (WM), and cerebrospinal fluid (CSF) masks. And the whole-brain averages (means of GM, WM, and CSF values) are reported in Fig. 4. Higher values indicate superior image quality. The estimation may be provided with only one foreground region in which the noise is computed as follows:
  $${\rm{SNR}}=\frac{{\mu }_{F}}{{\sigma }_{F}\sqrt{N/(N-1)}}$$
  where *μ*_*F*_ is the mean intensity of the foreground and *σ*_*F*_ is the standard deviation of the same region. *N* is the number of voxels in foreground mask.
(ii) Entropy Focus Criteria (EFC)^48^: EFC was derived from the Shannon entropy of voxel intensities relative to the maximum potential entropy for an image of similar size. Lower EFC indicates less ghosting and motion-induced blurring due to head movement during scanning, corresponding to enhanced image quality.
  $${\rm{EFC}}=\left(\frac{N}{\sqrt{N}}\log {\sqrt{N}}^{-1}\right){\rm{E}}$$
  where E is expressed as:
  $${\rm{E}}=-\mathop{\sum }\limits_{j=1}^{N}\frac{{x}_{j}}{{x}_{\max }}\mathrm{ln}\left[\frac{{x}_{j}}{{x}_{\max }}\right]$$
  Where *N* is the total number of voxels (i.e., independent voxels in the image), *x*_*j*_ represents the intensity value of the *j-th* voxel, typically the grayscale value or signal intensity of that voxel in the image. *x*_*max*_ is the total energy of all voxels, calculated as: ${x}_{\max }=\sqrt{\mathop{\sum }\limits_{j=1}^{N}{x}_{j}^{2}}$.

**Spatial anatomical Metrics:**

(iii) Contrast-to-Noise Ratio (CNR)^47^: Specific to structural MRI, CNR quantifies the distinguishability between tissue types (e.g., GM and WM). Higher CNR values indicate better tissue contrast.
  $${\rm{CNR}}=\frac{\left|{\mu }_{{\rm{GM}}}-{\mu }_{{\rm{WM}}}\right|}{\sqrt{{\sigma }_{B}^{2}+{\sigma }_{{\rm{WM}}}^{2}+{\sigma }_{{\rm{GM}}}^{2}}}$$
  where *σ*_*B*_ is the standard deviation of the noise distribution within the air (background) mask. *μ*_WM_ is the mean of signal within the WM mask, *μ*_GM_ is the mean signal within the GM mask. *σ*_WM_ is the standard deviation within the WM mask. *σ*_GM_ is the standard deviation within the GM mask.

**Temporal functional Metrics:**

(iv) Standardized DVARS^49^: This metric reflects the average voxel-wise change in signal intensity between consecutive rfMRI volumes, normalized with the standard deviation of the temporal difference time series. Lower standardized DVARS values indicate better image quality. For time point *t*:
  $${{\rm{DVARS}}}_{t}=\sqrt{\frac{1}{N}\sum _{i}{\left[{x}_{i,t}-{x}_{i,t-1}\right]}^{2}}$$
  Where *N* is the number of voxels and *x*_*i,t*_ is the signal intensity for voxel *i*.
(v) Mean Framewise Displacement (FD)^50^: FD estimates head motion by summing the absolute translational and rotational displacements across the x, y, and z axes for each time point. Lower values indicate less head motion.
  $${{\rm{FD}}}_{t}=\left|\Delta {d}_{x,t}\right|+\left|\Delta {d}_{y,t}\right|+\left|\Delta {d}_{z,t}\right|+\left|\Delta {\alpha }_{t}\right|+\left|\Delta {\beta }_{t}\right|+\left|\Delta {\gamma }_{t}\right|$$
  Where $\Delta {d}_{x,t}$, $\Delta {d}_{y,t}$, and $\Delta {d}_{z,t}$ are the translations along the x, y, and z axes, respectively, and $\Delta {\alpha }_{t}$, $\Delta {\beta }_{t}$, and $\Delta {\gamma }_{t}$ are the rotations around the x, y, and z axes, respectively.
(vi) Temporal SNR (tSNR)^51^: tSNR was computed as the mean BOLD signal intensity across the brain volume divided by its standard deviation within each voxel, across each run and for each subject. Higher values indicate better quality.
  $${\rm{tSNR}}=\frac{\langle S{\rangle }_{t}}{{\sigma }_{t}}$$
  where $\langle S{\rangle }_{t}$ is the average BOLD signal (across time), and ${\sigma }_{t}$ is the corresponding temporal standard-deviation map.

Figure 4 displays IQMs for T1-weighted, T2-weighted, and rfMRI data acquired at 3 T and 7 T field strengths. Except where indicated as non-significant (ns), all other within-modality comparisons between 3 T and 7 T showed statistically significant differences (Fig. 4a–c). Figures 4d–f demonstrate significant differences (p < 0.001) in DVARS and tSNR for rfMRI. Specifically, 7 T T2-weighted images generally exhibited significantly higher SNR than 3 T images, indicating improved overall signal quality. However, increased variance at 7 T suggests greater susceptibility to magnetic field inhomogeneities, potentially compromising signal quality in specific local regions compared to 3 T. EFC values remained relatively consistent across modalities, with 7 T T1-weighted images showing slightly better results, indicating lower image complexity and higher quality. For rfMRI, 3 T data exhibited a higher standard deviation of DVARS, reflecting greater motion artifacts. In contrast, 7 T data showed more stable inter-volume signal changes. Minimal differences in motion artifacts were observed between 3 T and 7 T rfMRI, as indicated by mean FD; however, 7 T displayed slightly lower values, suggesting less movement. Conversely, 3 T rfMRI showed significantly higher tSNR, reflecting greater signal stability over time.

## Usage Notes

The dataset, accessible via Figshare^43^, provides paired whole-brain T1-weighted, T2-weighted, and rfMRI scans acquired at both 3 T and 7 T field strength, along with manual hippocampal subfield annotations for the 7 T T2-weighted images. This unique combination of multi-contrast and multi-field strength data supports a wide range of machine learning applications.

The paired data enables diverse deep learning workflows, including cross-domain tasks such as 3T-to-7T image synthesis, which translates lower-resolution 3 T scans into high-resolution 7T-like images. This enhances image detail and quality for downstream applications. The high-quality manual annotations of hippocampal subfields on the 7 T T2-weighted images serve as reliable ground truth for training and evaluating segmentation models, particularly valuable given the importance of high-resolution detail in hippocampal substructures. This facilitates the development of advanced segmentation algorithms applicable to high-resolution imaging and studies of hippocampal morphology in health.

The multi-modality nature of the dataset further allows for multi-modal research workflows integrating structural and functional brain imaging. Researchers can explore relationships between brain anatomy and functional connectivity by aligning data across T1-weighted, T2-weighted, and rfMRI modalities, enabling joint representation learning and in-depth investigation of brain structure-function interactions. This makes the dataset particularly well-suited for multi-modal deep learning applications.

While this dataset offers a valuable benchmark for comparing 3 T and 7 T imaging, it’s crucial to acknowledge its limitations. The relatively small sample size, comprising only healthy young adults, restricts the generalizability of models trained solely on this dataset. Data augmentation is therefore recommended, particularly for deep learning applications. Furthermore, researchers studying disease or diverse age groups should supplement this dataset with additional data to enhance model robustness. Despite these limitations, the dataset provides a critical baseline for understanding fundamental hippocampal structure before considering age- or disease-related variations.

Clinical applications of generated images require caution due to potential anatomical inaccuracies. While these images can augment existing imaging modalities, they should not replace established diagnostic procedures but serve as supplementary tools to enhance diagnostic insights, especially when conventional high-resolution images are unavailable. Furthermore, continuous validation and assessment processes are necessary to ensure reliability and accuracy.

Furthermore, several technical limitations inherent to 7 T MRI must be considered. Although 7 T MRI enhances signal and contrast, challenges like field inhomogeneities and signal variability need to be acknowledged, especially in precise structural imaging. In-plane dephasing and signal loss at tissue-air interfaces can introduce artifacts, particularly when imaging structures near the skull base, like the temporal lobe. As shown in Fig. 1, substantial intensity variations and loss, particularly in the temporal lobe of 7 T T2-weighted images, result from susceptibility effects and magnetic field inhomogeneity, especially at gray matter-CSF boundaries. While these artifacts minimally affect hippocampal visualization and segmentation (the dataset’s primary focus), they can impact overall image quality and potentially affect whole-brain image generation. Users are advised to exercise caution when using whole-brain data for segmentation or generative tasks, as signal loss may occur in specific regions, particularly in the temporal lobes on 7 T T2-weighted images for certain subjects. The absence of 7 T DWI sequences reflects common technical constraints at 7 T, including shorter relaxation times, increased magnetic field inhomogeneity, and higher SAR, which prevented the acquisition of high-quality DWI data with our scanner and available sequences/software.

Therefore, users should critically assess the impact of sample size and imaging artifacts when interpreting results to ensure that the conclusions drawn align with the dataset’s scope and limitations. However, we emphasize that the high-quality manual annotations, consistent protocols, and paired 3T-7T scans offer a valuable resource for early-stage model development and evaluation. Future dataset extension could include larger, more diverse samples, encompassing pathological cases, to improve generalizability.

## Data Availability

The brain MRI data was processed using the following open-source software packages: BIDScoin (version 4.3.0), BIDS-validator, MRIQC (version 22.0.6). No custom code has been used.
